# Antenatal Corticosteroid Therapy Attenuates Angiogenesis Through Inhibiting Osteoclastogenesis in Young Mice

**DOI:** 10.3389/fcell.2020.601188

**Published:** 2020-12-15

**Authors:** Yu Chai, Jianwen Su, Weisheng Hong, Runjiu Zhu, Caiyu Cheng, Lei Wang, Xianrong Zhang, Bin Yu

**Affiliations:** ^1^Division of Orthopaedic Surgery, Department of Orthopaedics, Nanfang Hospital, Southern Medical University, Guangzhou, China; ^2^Guangdong Provincial Key Laboratory of Bone and Cartilage Regenerative Medicine, Nanfang Hospital, Southern Medical University, Guangzhou, China

**Keywords:** ACT, angiogenesis, osteoclastogenesis, EZH2, pre-osteoclasts

## Abstract

Antenatal corticosteroid therapy (ACT) has been shown to reduce morbidity and mortality rates in preterm delivery, but the fetus is more likely to face the risk of low bone mineralization and low fetal linear growth. However, the mechanism of ACT inducing low bone mineralization remains largely unknown. Pre-osteoclasts, which play an important role in angiogenesis and osteogenesis, are specifically regulating type H vessels (CD31^hi^Emcn^hi^) and vessel formation by secreting platelet-derived growth factor-BB (PDGF-BB). We find that the number of pre-osteoclasts and POC-secreted PDGF-BB is dramatically decreased in ACT mice, contributing to the reduction in type H vessels and bone mineralization during the mouse offspring. Quantitative analyses of micro-computed tomography show that the ACT mice have a significant reduction in the mass of trabecular bone relative to the control group. Mononuclear pre-osteoclasts in trabecular bone decreased in ACT mice, which leads to the amount of PDGF-BB reduced and attenuates type H vessel formation. After sorting the Rank+ osteoclast precursors using flow cytometry, we show that the enhancer of zeste homolog 2 (Ezh2) expression is decreased in Rank+ osteoclast precursors in ACT mice. Consistent with the flow data, by using small molecule Ezh2 inhibitor GSK126, we prove that Ezh2 is required for osteoclast differentiation. Downregulating the expression of Ezh2 in osteoclast precursors would reduce PDGF-BB production. Conditioned medium from osteoclast precursor cultures treated with GSK126 inhibited endothelial tube formation, whereas conditioned medium from vehicle group stimulated endothelial tube formation. These results indicate Ezh2 expression of osteoclast precursors is suppressed after ACT, which reduced the pre-osteoclast number and PDGF-BB secretion, thus inhibiting type H vessel formation and ACT-associated low bone mineralization.

## Introduction

Antenatal corticosteroid therapy (ACT) is widely used for several types of pregnancy disorders, including preparing the fetal lung for impending preterm birth, congenital adrenal hyperplasia, multiple pregnancies, and placenta previa (Young et al., 1980; Morales et al., 1986; Bloom et al., 2001; Maryniak et al., 2014), but animal and human studies link corticosteroids to smaller birth size and suppression of fetal bone turnover at birth in corticosteroid-exposed infants (Bloom et al., 2001; Murphy et al., 2008). Even more, a randomized clinical trial shows strong evidence that lower birth weight is highly relative to lower peak bone mass in ACT patients (Godfrey et al., 2011). Our previous researches show that antenatal corticosteroid exposure could impair bone development and angiogenesis and reduce the peak bone mass during the adult offspring (Sliwa et al., 2010; Cheng et al., 2014; Zhang et al., 2016; Chen et al., 2018; Su et al., 2020). The mechanism underlying ACT suppression of angiogenesis and osteogenesis in relation to lower peak bone mass and growth retardation remains unknown. Pre-osteoclasts, the immature osteoclast precursor cells, are well known to promote angiogenesis of type H vessels through the release of platelet-derived growth factor-BB (PDGF-BB) (Xie et al., 2014). Angiogenesis is coupling with the osteogenesis, participating in bone modeling and remodeling during the whole lifespan (Brandi and Collin-Osdoby, 2006; Seeman, 2009; Teti, 2011). During new vessel growth, the concentration of angiogenesis factors, such as PDGF-BB from pre-osteoclasts, in local environment must be secreted precisely to induce formation of new vessels (Bronckaers et al., 2014; Nassiri and Rahbarghazi, 2014). Corticosteroids are known to inhibit angiogenesis and decrease pre-osteoclasts (Buckley and Humphrey, 2018), suggesting that corticosteroids may impact pre-osteoclasts secreting angiogenesis factors, which support angiogenesis (Pufe et al., 2003; Weinstein, 2010; Yang et al., 2018).

Enhancer of zeste homolog 2 (EZH2), which is the methyltransferase subunit of the polycomb repressive complex 2 (PRC2), deposits the heterochromatic mark H3K27me3 that silences gene expression (Margueron and Reinberg, 2011). Evidence from different groups demonstrates that Ezh2 plays an important role in bone growth and bone remodeling (Dudakovic et al., 2015). Ezh2 was shown to epigenetically repress the osteoclast inhibitory factor IRF8, and Ezh2 also can play epigenetic and cytoplasmic roles during osteoclast differentiation by suppressing MafB transcription and regulating early phases of RANKL signaling (McCabe et al., 2012; Dudakovic et al., 2016; Fang et al., 2016; Hemming et al., 2017). Because osteoclastogenesis can be mediated by the Ezh2, we explored the Ezh2 playing a critical role in osteoclastogenesis during ACT.

Therefore, in this study, we further investigated the role of Ezh2-mediated osteoclastogenesis during the ACT. We demonstrated that antenatal corticosteroid exposure decreases osteoclastogenesis and angiogenesis by suppressing Ezh2 expression in osteoclast precursors, which cause the pre-osteoclast number and pre-osteoclast-derived PDGF-BB decrease in bone, leading to the reduced angiogenesis and bone mass in bone.

## Results

### ACT Decreases Bone Volume and Angiogenesis in Long Bone

**FIGURE 1:**
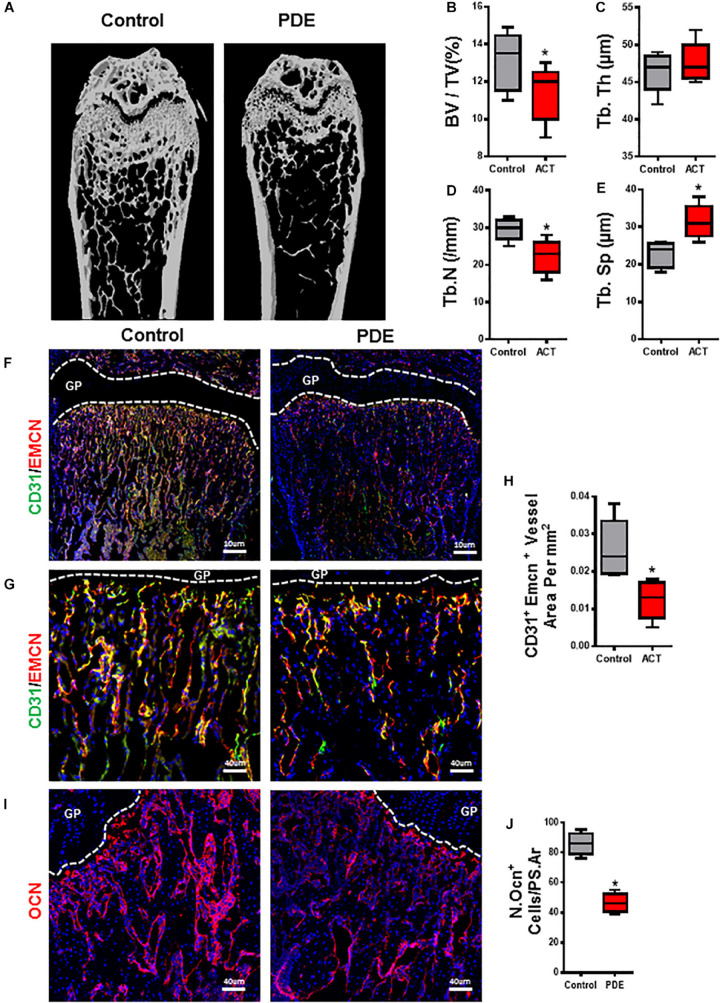
Prenatal dexamethasone exposuredecreases bone volume and angiogenesis in long bone. Representative μCT images of distal femur in 12-week-old female mouse offspring are shown in panel **(A)**. Quantitative analyses of femoral microstructural parameters of female mouse offspring including trabecular bone volume fraction (BV/TV) **(B)**, trabecular number (Tb. N) **(C)**, trabecular thickness (Tb. Th) **(D)**, and trabecular separation (Tb. Sp) **(E)**. **(F,G)** Representative images of double-immunofluorescence staining for CD31 (green) and endomucin (Emcn, red) in femoral metaphysis from 12-week-old female offspring. DAPI stains nuclei blue. Images in panel **(F)** are lower power with boxes outlining the area of higher power in panel **(G)**. Quantification of the relative fluorescence area of CD31^+^Emcn^+^ cells per tissue area in femoral metaphysis (CD31^+^Emcn^+^ area per mm^2^) **(H)**. **(I)** Representative images of immunofluorescence staining for osteocalcin (red) and **(J)** quantitative analysis of osteocalcin^+^ cells in femoral metaphysis from 12-week-old female offspring. DAPI stains nuclei blue. *p < 0.05.

Bone modeling and remodeling are coupling with angiogenesis and osteogenesis by a specific vessel subtype, type H vessel, during offspring (Kusumbe et al., 2014; Ramasamy et al., 2014). Our group’s previous finding has shown the adverse effect of ACT on the long bone in fetal mice. We then tested whether long bone osteogenesis and angiogenesis are being retreated or not. Femora were harvested from 12-week-old mouse of ACT, and bone architecture of the femoral bone was measured by micro-computed tomography (μCT). A significant reduction in the mass of trabecular bone was observed in ACT mice relative to control mice (Figure 1A). The ACT mice exhibited reduced trabecular bone volume (BV/TV) and number (Tb. N) and greater trabecular bone separation (Tb. Sp) compared with control littermates in female mice (Figures 1B,D,E). No significant differences were observed in trabecular thickness (Tb. Th) (Figure 1C). Together, these data revealed that ACT has adverse effects on bone architecture.

We then detected type H vessels (CD31^hi^Emcn^hi^), which have been recognized as osteogenesis-coupling neovessels responsible for new bone formation in femur of ACT mice. A decrease in CD31^hi^Emcn^hi^ blood vessels in primary spongiosa in femora of ACT mice (Figures 1F–H) is detected by immunofluorescence staining relative to control mice. A similar reduction in bone surface OCN+ osteoblasts in the same region was also observed in the ACT mice (Figures 1I,J). Therefore, ACT reduces type H vessel, which is highly associated with impaired bone formation in mouse offspring.

### Pre-osteoclast Number and Its Synthesis of PDGF-BB Decreased in ACT Mice

**FIGURE 2:**
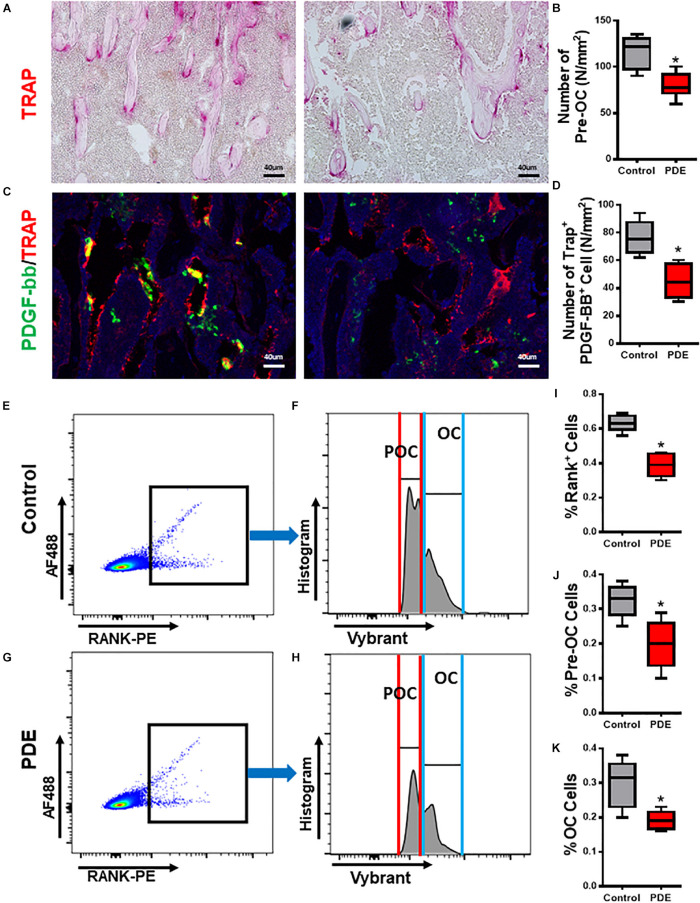
Pre-osteoclast number and its synthesis of PDGF-BB decreased in ACT mice. **(A,C)** Representative TRAP staining (magenta) images **(A)** and immunofluorescence images of TRAP (red) and PDGF-BB (green) costaining (yellow) **(C)** in femoral metaphysis from 12-week-old female offspring. DAPI stains nuclei blue. Scale bar = 40 μm. **(B,D)** Quantitative analysis of the N. POCs **(B)** and the N. TARP^+^ PDGF-BB^+^ cells **(D)** in distal femur. **(E–H)** Representative flow cytometry plots showing the sorting profile used to separate POCs and OCs in femoral bone from 12-week-old female mouse offspring. RANK^+^ cells with 1-2 nuclei (POCs) and greater than three nuclei (OCs) are sorted for gene expression analysis. **(I–K)** Quantitative analysis of the percentage of RANK^+^ cells **(I)**, POCs **(J)**, and OCs **(K)** in distal femur. *p < 0.05.

It has been previously reported that pre-osteoclasts produced PDGF-BB to couple angiogenesis and osteogenesis during bone modeling and remodeling. Excessive GCs could decrease pre-osteoclasts and its synthesis of PDGF-BB (Andrae et al., 2008; Xie et al., 2014). In adult glucocorticoid-induced osteoporosis mouse models, osteoclasts were shown to have increased resorption in early phase due to enhanced survival of osteoclasts, but suppression of bone resorption in late phase due to the glucocorticoid suppression of osteoclast differentiation (Jia et al., 2006). But how ACT shows effects on osteoclastogenesis in ACT mice remains unknown. We examined the effect of ACT on the number of pre-osteoclasts and synthesis of PDGF-BB. The number of mononuclear Trap+ pre-osteoclasts was significantly decreased in 12-week-old ACT mice relative to the control mice (Figures 2A,B). To further document the loss of pre-osteoclast number with ACT, Rank+ osteoclast precursors were analyzed by flow cytometry and found dramatically decreased in ACT group relative to the control mice (Figures 2E–J). Immunofluorescence staining showed that the double-positive staining (TRAP^+^. PDGF-BB+) percent was decreased in ACT mice relative to the control mice by 12 weeks old (Figures 2C,D). Overall, our results indicate that ACT could inhibit osteoclastogenesis and decrease PDGF-BB secretion from pre-osteoclast.

### Prenatal Dexamethasone Exposure Decreases Ezh2 Expression in Osteoclast Precursors and Attenuates Osteoclastogenesis

**FIGURE 3:**
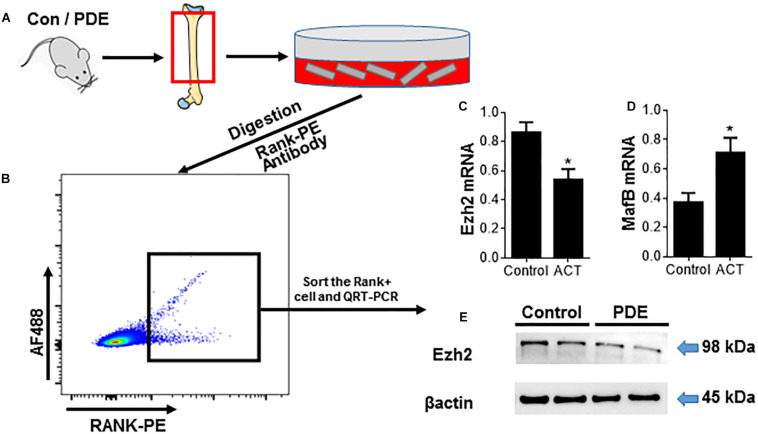
Prenatal dexamethasone exposure decreases Ezh2 expression in osteoclast precursors and attenuates osteoclastogenesis. **(A)** Diagram showing the procedure for the isolation of OCs from femoral metaphysis (see the detailed description in section “Materials and Methods”). **(B–E)** RANK+ cells were isolated from femoral metaphyses using the same method as in Figure 2. **(B)** Representative flow cytometry plots showing the sorting profile used to separate POCs and OCs in femoral bone from 12-week-old female mouse offspring. Quantitative RT-PCR analyses of Ezh2 **(C)** and MafB **(D)** mRNA expression and Western blot analysis of Ezh2 **(E)** protein expression in the sorted cells are shown. β-Actin serves as control to ensure appropriate loading. *p < 0.05.

Enhancer of zeste homolog 2 has been mentioned playing an important role in osteoclast differentiation by silencing the negative regulator IRF8 (Fang et al., 2016) and plays epigenetic and cytoplasmic roles during osteoclast differentiation by suppressing MafB transcription and regulating early phases of RANKL signaling. To investigate whether the effect of ACT on osteoclast differentiation is being mediated by Ezh2, we sorted the Rank+ osteoclast precursors and analyzed the Ezh2 expression (Figures 3A,B). The number of Rank+ osteoclast precursors decreased in the ACT mice group relative to the control mice (Figures 2I–K), and Ezh2 mRNA expression level (Figure 3C) and protein level (Figure 3E) were decreased; MafB mRNA level was upregulated in ACT mice (Figure 3D).

### Ezh2 Plays a Critical Role in Osteoclastogenesis by Activating Osteoclast Regulators and Represses the Osteoclast Inhibitory Factors

**FIGURE 4:**
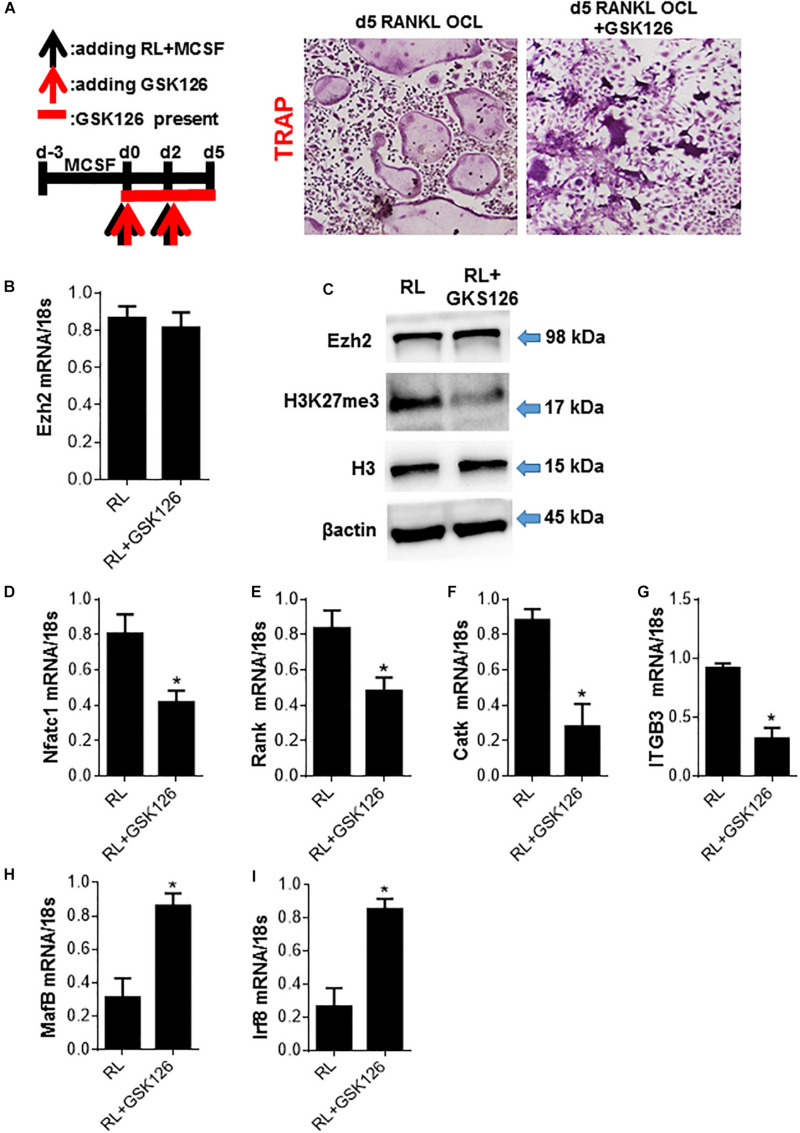
Ezh2 plays a critical role in osteoclastogenesis by activating osteoclast regulators and repress the osteoclast inhibitory factors. BMMs were cultured with MCSF for 3 days (days 3 to 0) to form OCL precursors (OCLp) before addition of RANKL/MCSF at day 0 to generate OCL. **(A)** OCLp were cultured with RANKL/MCSF in the presence of the indicated concentrations of GSK126 for 5 days. TRAP+ OCL formed were analyzed by microscopy (representative images on left) and the counts for multinucleated (*n* ≥ 3) TRAP+ OCL/well graphed (right). GSK126 (10 μM) was added at the indicated time intervals during the processes of OCLp and OCL (day 4) formation, and representative images and counts for multinucleated (*n* ≥ 3) TRAP+ OCL/well are shown. **(B)** OCLp (day 0) or stimulated with RANKL/MCSF ± GSK126 (10 μM; GSK) for 5 days were analyzed for Ezh2 mRNA by quantitative PCR (qPCR). **(C)** OCLp cultures (day 0) or after stimulation with RANKL/MCSF ± GSK126 (10 μM) for 1 day were analyzed for total cellular levels of Ezh2, H3K27me3, H3, and β-actin by WB. **(D–G)** OCLp (day 0) or after stimulation with RANKL/MCSF ± GSK126 (10 μM) for 5 days were analyzed for the expression of OCL-specific genes NFATc1, RANK, CatK, ITGB3 by qPCR. **(H,I)** OCLp (day 0) or after stimulation with RANKL/MCSF ± GSK126 (10 μM) for 1 day were analyzed for the expression of OCL-negative regulators MafB and Irf8 by qPCR. WB analyses show representative of three independent experiments and error bars in mRNA experiments represent SEM for 4 biological replicates. *p < 0.05.

To further demonstrate whether Ezh2 plays an important role in osteoclastogenesis, we treated primary bone marrow-derived monocytes (BMMs) (osteoclast precursors) with Ezh2 methyltransferase domain inhibitor GSK126. We showed the inhibitory effects of GSK126 on RANKL-induced differentiation of Trap+ multinucleated osteoclasts (Figure 4A). To our surprise, GSK126 did not alter the Ezh2 mRNA and protein levels (Figures 4B,C), but effectively decreased the cellular levels of H3K27me3 (Figure 4C). Consistent with other research finding, GSK126 reduced RANKL-mediated upregulation of critical early osteoclast regulators NFATC1, Rank, Catk, and ITGB3 (Figures 4D–G). These results support a role for Ezh2 in osteoclast differentiation, and GSK126 could suppress osteoclastogenesis by inhibiting Ezh2 catalytic activity. Furthermore, effective osteoclastogenesis also requires downregulating of transcriptional repressors, such as IRF8, BCL6, or Mafb, which is against the differentiation program. GSK126 could prevent the downregulation of osteoclast inhibitory factors Mafb and Irf8 (Figures 4H,I). Together, we find that Ezh2 plays a critical role in osteoclastogenesis and mediates repression of OCL-negative regulators.

### Loss of Ezh2 Suppresses Osteoclast Precursors PDGF-BB Expression and Bone Vasculature in ACT Mice

**FIGURE 5:**
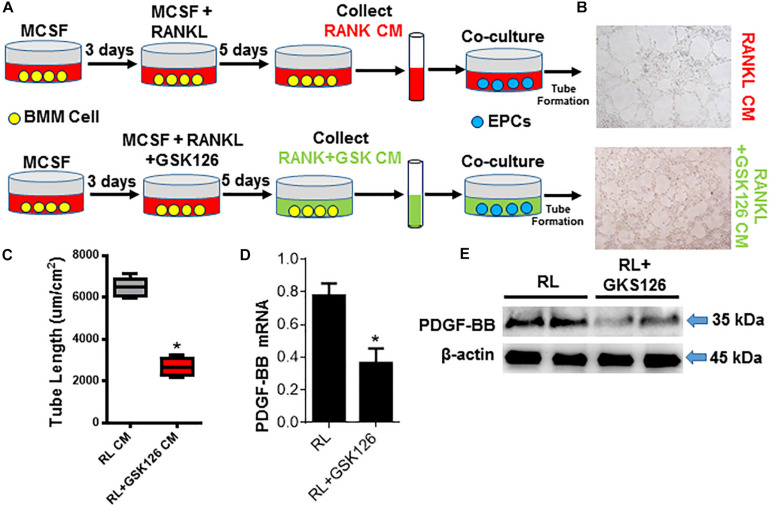
Loss of Ezh2 suppress osteoclast precursors PDGF-BB expression and bone vasculature in ACT mice. **(A)** Diagram showing the procedure for the collection of condition media of OCs under denoted conditions and the co-culture with the EPCs. **(B,C)** Representative images **(B)** and tube length **(C)** from Matrigel endothelial tube formation assay with addition of OCLp (red) and after stimulation with RANKL/MCSF ± GSK126 (10 μM) (green) culture media. **(D,E)** Quantification of Pdgfb mRNA by real-time PCR **(D)** and total cellular levels of PDGF-BB and β-actin by WB **(E)**. mRNA normalized to GAPDH. *p < 0.05.

It has been proved that pre-osteoclasts are the major source of PDGF-BB. And in the ACT mouse model, Ezh2 expression was dramatically decreased in osteoclast precursors and attenuated the osteoclastogenesis process. Not only the number of pre-osteoclast, but also the PDGF-BB protein level was decreased in Trap+ mononuclear pre-osteoclasts of ACT mice. In order to investigate the effect of Ezh2 on osteoclast-mediated coupling angiogenesis in ACT mouse model, we perform a cotreated experiment. After treating the osteoclast precursors with GSK126 and MCSF/RANKL, the PDGF-BB mRNA level and protein level were decreased in osteoclast precursors (Figures 5D,E). Matrigel tube formation assays with endothelial precursor cells plus GSK126 POC-conditioned medium demonstrated suppressed tube formation rate relative to the control POC-conditioned medium (Figures 5A–C). Together, ACT disrupts angiogenesis and osteogenesis coupling by repression Ezh2 expression in POC and causes POC number, and POC-secreted PDGF-BB dramatically decreased, which was associated with low bone mass and growth retardation in ACT mice.

## Discussion

Multiple doses of glucocorticoids are in widespread use in obstetric practice to accelerate the maturation of fetal cardiovascular systems when facing preterm delivery, as well as to provide treatment for congenital adrenal hyperplasia of the fetus or asthma of pregnant women (Maryniak et al., 2014). Prenatal dexamethasone exposure has persisting “programming” effects on offspring in rodents and other model species (de Vries et al., 2007). ACT could impair bone development and angiogenesis and reduces the peak bone mass during the adult offspring (Godfrey et al., 2011). The mechanism underlying ACT suppression of angiogenesis and osteogenesis in relation to lower peak bone mass and growth retardation remains unknown. Because the dose of dexamethasone used in human pregnancy varies from 0.02 mg⋅kg^–1^ per day for congenital adrenal hyperplasia (Meyer-Bahlburg et al., 2004) to 0.5 mg⋅kg^–1^ per day for indicated preterm labor (Bloom et al., 2001; Peaceman et al., 2005), it is important to use a proper dose of dexamethasone. In animal research, a much lower therapeutic dose of dexamethasone (0.03 mg⋅kg^–1^ every second day) could induce loss of trabecular bone and retardation of bone growth in developmental piglets (Tomaszewska et al., 2013). Our previous work found that the intrauterine growth retardation rate is dose-dependent (Xu et al., 2011). Dose conversion between mice and human (conversion factor is 0.08) (Reagan-Shaw et al., 2008) and prenatal treatment with dexamethasone in mice at (1.2 mg⋅kg^–1^ per day) in this study are comparable with those prescribed for pregnant women (0.1–0.2 mg⋅kg^–1^). Recent studies indicated that prenatal dexamethasone treatment at (1 mg⋅kg^–1^ per day) induced an adverse effect on long bone development. We undertook this study to investigate the effect of prenatal dexamethasone exposure on fetal bone development and clarify the underlying mechanisms. Our data show that ACT treatment could impact the osteoclastogenesis, angiogenesis, and osteogenesis during bone development by reducing Ezh2 expression in osteoclast precursors, which plays a critical role in osteoclast precursors differentiating into pre-osteoclast. With the decreased number of pre-osteoclasts of ACT mice, the PDGF-BB protein, secreted by pre-osteoclasts, dramatically decreased and impacted the type H vessel formation.

Angiogenesis is coupled with bone formation in these processes for proper bone homeostasis (Brandi and Collin-Osdoby, 2006; Portal-Nunez et al., 2012); reduced angiogenesis impacts the osteogenesis by decreasing BNM transport nutrients, oxygen, minerals, and metabolic wastes, which are essential for maintaining proper osteoblastic bone matrix synthesis and mineralization (Choi et al., 2002; Percival and Richtsmeier, 2013). During the bone modeling and remodeling, osteoclast bone resorption is coupled with osteoblast bone formation. Endocrine and paracrine factors from osteoblast and osteoclast could induce migration and differentiation of the osteoblast/osteoclast precursors. In response to stimulation with MCSF and RANKL, macrophages and monocytes commit to the osteoclast lineage as TRAP^+^ mononuclear cells, which is defined as pre-osteoclast (Del Fattore et al., 2012). Pre-osteoclasts secrete PDGF-BB to stimulate migration and angiogenesis of endothelial progenitor cells (EPCs) and MSCs (Fiedler et al., 2004; Wang et al., 2012). Pre-osteoclasts subsequently fuse to form TRAP^+^ multinuclear cells (osteoclasts). Growth factors, transforming growth factor-β, and insulin-like growth factor-1, are released from the bone matrix during osteoclast bone resorption to recruit BMSCs to the same zone, differentiated into osteoblast for new bone formation.

It is being increasingly proven that histone methylation of bivalent promoters in central to the progression of RANKL-induced osteoclast differentiation by both activating (Yasui et al., 2011; Nakamura et al., 2017) and repressing (Fang et al., 2016) selected important gene promoters in osteoclast precursors. Ezh2-directed H3k27me3 chromatin repression of developmental genes is a key to the cellular differentiation and cellular senescence (Margueron and Reinberg, 2011; Li et al., 2017). Recent reports have shown that Ezh2 could regulate a subset of anti-inflammatory and pro-osteoclastogenic genes during interferon-γ-mediated macrophage activation. Also Ezh2 activity and total cellular H3K27me3 levels are upregulated during the initial 12 to 24 h of RANKL-induced pre-osteoclasts (Adamik et al., 2020). Ezh2 could repress the expression of osteoclast inhibitory factors MafB, Arg1, and Irf8. In our study, we demonstrated that ACT could repress the Ezh2 expression in osteoclast precursors and attenuated the osteoclastogenesis in ACT mice. The MafB and Irf8 mRNA expression level is upregulated after treating osteoclast precursors with GSK126 (Ezh2 specific inhibitor), which represses the osteoclastogenesis. To our knowledge, it is the first time that we find the pre-osteoclasts and PDGF-BB derived from pre-osteoclast decreased in the ACT mice model and caused the type H vessel decrease, which leads to dysfunction of osteogenesis. Nevertheless, a limitation of our study that cannot be neglected is that we failed to explore the mechanism of Ezh2 downregulated by ACT.

Therefore, further study is necessary to unravel the cellular and molecular mechanisms of how ACT regulates Ezh2 expression in osteoclast precursor.

## Materials and Methods

### Animals

This study was conducted in accordance with the Guide for the Care and Use of Laboratory Animals of Nanfang Hospital Southern Medical University. The protocol was approved by the Animal Care and Use Committee of Nanfang Hospital. Pathogen-free mice were maintained under standard conditions in a 12-h light and 12-h dark cycle, at 25°C ± 3°C, with a relative humidity of 40 to 60% and with food and tap water available *ad libitum*. ACT during GD 12 to 14 was applied according to the procedure previously described (Chen et al., 2018). Briefly, virgin C57BL/6 female mice at 10 to 12 weeks old were mated with male mice overnight. The day of the presence of a vaginal plug was set as GD 0; the pregnant mice were randomly assigned to the ACT group or vehicle treatment (control) group. To construct ACT mice model, dexamethasone sodium phosphate (Cat. 2392-39-4, Tianxin, China) was injected subcutaneously (1.2 mg kg^–^1 per day) during GD 12 to 14. To construct the vehicle control of ACT model, pregnant mice were treated with the same amount of vehicle (normal saline) daily during GD 12–14. The pregnant mice were housed individually in cages with freely available food and water. Two female offspring were selected randomly from each litter for postnatal bone development investigation. Femora from female mice offspring at 12 weeks old were dissected for further analysis.

### μCT Analysis

Femora from 12-week-old mice offspring were dissected free of soft tissue, fixed and stored in 70% ethanol, and imaged using a μCT specimen scanner (Scanco Medical, AG, Switzerland). The scan was performed using an X-ray energy of 55 kVp and current of 145 mA, with a voxel size of 9 μm and an integration time of 400 ms. Cross-sectional images of the distal femur were used to perform three-dimensional histomorphometric analysis of trabecular bone. The sample area selected for analyses was of 2-mm length of the metaphyseal secondary spongiosa, originating 1.0 mm below the epiphyseal growth plate and extending caudally.

### Histochemistry

Femora of mice offspring at 12 weeks old were fixed in 4% paraformaldehyde, decalcified in 0.5 M ethylenediaminetetraacetic acid (pH 8.0), and then followed by paraffin embedding or frozen embedding. Serial longitudinal sections (4 μm thick) were cut. For detecting the alkaline phosphatase activity in bone, TRAP staining was performed on the deparaffinized and rehydrated sections using a leukocyte alkaline phosphatase kit (Cat. 387A-1KT, Sigma-Aldrich, United States). The TRAP^+^ multinucleated cells containing at least three nuclei were identified as osteoclasts under light microscope (Olympus, BX53). The TRAP^+^ cells per square millimeter of metaphyseal area (N. mm^–2^) in the area from 0 to 0.5 mm below growth plate were quantified.

### Immunofluorescence

For immunofluorescence staining, frozen sections were incubated in blocking buffer [3% bovine serum albumin in phosphate-buffered saline (PBS) with Triton X-100 (PBST)] for 1 h at room temperature and incubated with primary antibodies overnight at 4°C. The primary antibodies for immunostaning include osteocalcin (M173, Takara, 1:200), CD31 (FAB3629G-100, R&D Systems, 1:100), endomucin (Emcn, SC-65495, Santa Cruz, 1:100), and PDGF-BB (sc-365805, Santa Cruz, 1:100). Sections were washed three times in PBS and then incubated with secondary antibodies for 1 h at room temperature. The secondary antibodies for immunostaning include 488-conjugated secondary antibody (703-546-155, Jackson ImmunoResearch, 1:200) and 594-conjugated secondary antibody (712-586-153, Jackson ImmunoResearch, 1:200). Nuclei were counterstained with DAPI (S2110, Solarbio, China). Images were captured using a fluorescence microscope (Olympus, BX53, Japan). Positively stained area or relative staining intensity was measured in the area from 0 to 0.5 mm below growth plate.

### Cell Sorting and Flow Cytometry Analysis

For flow cytometric analysis and sorting of mouse RANK^+^ MSPCs from femoral metaphysis, we dissected the femora free of soft tissues from mice offspring at 12 weeks old. The epiphysis was removed, and the bones were cut into small pieces and digested in the protease solution for 20 min. Cells within the supernatant were collected for flow cytometry. Cell numbers were determined after removal of red blood cells with ACK Lysis Buffer (CS0001, Leagene, China). Cells were then sorted according to side scatter and RANK-PE fluorescence at >10^3^ log Fl-1 (RANK-PE) fluorescence. FACS was performed using a 5-laser Beckman FACS system (MoFlo XDF, Beckman, United States). Flow cytometric analyses were performed using FlowJo software (BD Life Sciences, San Jose, CA, United States). The primary antibody used was RANK-PE (12-6612-82, Thermo Fisher Scientific, 5 μL/10^6^ cells).

### Western Blotting

Western blotting was performed according to standard protocols using the following antibodies directed toward Ezh2 (5246S; Cell Signaling Technology), GAPDH (ET1601-4; HuaBio), PDGF-BB (sc-365805, Santa Cruz), and β-actin (85316, Sigma). Nuclear and cytoplasmic protein separations were performed using the Nuclear Extract Kit (Active Motif, 40010). Signals were developed by ECL (MilliporeSigma, Burlington, MA, United States) and visualized by Tanon chemiluminescence apparatus. Protein densitometry was quantified by ImageJ software (National Institutes of Health, Bethesda, MD, United States).

### OCL Differentiation and TRAP Staining

Primary BMM cells were expanded in αMEM with MCSF (20 ng/mL) (R&D Systems, Minneapolis, MN, United States) for 3 days (days 3 to 0) to generate OCLp. Then RANKL (10 ng/mL) (R&D Systems) and MCSF (10 ng/mL) were used at day 0 to differentiate OCL for 4 to 5 days. In some cases, BMMs were transduced with lentivirus particles for the first day (days 3 to 2) of MCSF generation of OCLp. OCL was fixed with 37% formaldehyde for 15 min, washed with ddH_2_O, and stained using leukocyte acid phosphatase kit (Sigma-Aldrich, 387A-1KT) following the manufacturer’s instructions. TRAP^+^ multinucleated cells with three or more nuclei were counted as OCL. All OCL formation experiments were scored from four to five independent wells.

### Quantitative Real-Time Polymerase Chain Reaction

POCs were incubated with prednisolone 10^–6^ M alone or with either RU486, GRsiRNA, or tumor necrosis factor α or prednisolone 10^–7^ M alone for 24 h. Total RNA for quantitative real-time (qRT)-polymerase chain reaction (PCR) was extracted from the cells using Trizol reagent (Invitrogen, Carlsbad, CA, United States) according to the manufacturer’s protocol. RNA purity was tested by measuring the absorbance at 260 and 280 nm. For qRT-PCR, cDNA was prepared with random primers using the SuperScript First-Strand Synthesis System (Invitrogen) and analyzed with SYBR Green Master Mix (Qiagen, Valencia, CA, United States) in the thermal cycler with two sets of primers specific for each targeted gene. Relative expression was calculated for each gene by the 2^–ΔΔCt^ method, with GAPDH for normalization. The quantitative PCR primers are listed in Table 1.

**TABLE 1:** qPCR primers for mouse mRNA analysis.

Gene	Forward primer (5′→3′)	Reverse primer (5′→3′)
mEzh2	TACATCCCTTCCATGCAACA	TCCCTCCAGATGCTGGTAAC
mNfatc1	TGGAGAAGCAGAGCACAGAC	GCGGAAAGGTGGTATCTCAA
mRank	GTGGAAATAAGGAGTCCTCAG	CACCGTCTTCTGGAACCATC
mCatk	AATACGTGCAGCAGAACGGA GGC	CTCGTTCCCCACAGGAATCT CTCTGTAC
mMafB	GAGCAGGTGTGACTCACGAT	TGGCTAGTGGGTAGCTGTTG
mIrf8	CTACCTGACAGCAGGGTGGT	GGCATACAGCTGCTCTACCTG
mPdgf-bb	ACCCAGAAGACTGTGGATGG	CACATT-GGGGGTAGGAACAC
mItgb3	CCACACGAGGCGTGAACTC	CTTCAGGTTACATCGGGGTGA

### *In vitro* Tube Formation Assay

Culture media was collected from POC cultures in the presence of RANKL (10 ng/mL) (R&D Systems) and MCSF (10 ng/mL) or in addition to GSK126 (Selleck; S7061) for 5 days. In a separate culture, EPCs were seeded in endothelial progenitor outgrowth cell growth medium (BioChain, Newark, CA, United States; Z7030033). Tube formation assays were performed by using Matrigel Matrix basement membrane (Corning, Corning, NY, United States; 356230) as reported. Briefly, 50 μL of Matrigel was added into 96-well culture plates and incubated at 37°C for 30 min to allow the gel to solidify. We then seeded EPCs (2 × 10^4^ cells/well) on polymerized Matrigel in plates and cultured the cells with the collected POC conditioned medium mixed with the endothelial cell culture medium in a ratio of 1:1. Two groups of cells were incubated in medium with RANKL and MCSF or additional GSK126. After incubation at 37°C for 4 h, we observed tube formation by microscopy and measured the cumulative tube lengths.

### Statistics

All quantitative data were presented as mean ± SEM. For comparisons between two groups, independent Student *t* test was performed. For multiple comparisons, one-way analysis of variance with Bonferroni *post hoc* test was used. Statistical analysis was performed using SPSS, version 20 software (IBM Corp.). Significant level was defined as *P* < 0.05.

## Data Availability Statement

The raw data supporting the conclusions of this article will be made available by the authors, without undue reservation.

## Ethics Statement

The animal study was reviewed and approved by Animal Care and Use Committee of Nanfang Hospital.

## Author Contributions

BY and XZ designed the research and drafted, revised, and approved the manuscript. YC and JS acquired the data. RZ, CC, and WH analyzed the data. All authors read and approved the final manuscript.

## Conflict of Interest

The authors declare that the research was conducted in the absence of any commercial or financial relationships that could be construed as a potential conflict of interest.
